# Removal and biodegradation of different petroleum hydrocarbons using the filamentous fungus *Aspergillus* sp. RFC‐1

**DOI:** 10.1002/mbo3.619

**Published:** 2018-03-25

**Authors:** Adnan B. Al‐Hawash, Xiaoyu Zhang, Fuying Ma

**Affiliations:** ^1^ Key Laboratory of Molecular Biophysics of MOE College of Life Science and Technology Huazhong University of Science and Technology Wuhan China; ^2^ Ministry of Education Directorate of Education Basra Iraq

**Keywords:** *Aspergillus*, bioaccumulation, biodegradation, degradation kinetics, petroleum hydrocarbons, removal

## Abstract

Petroleum pollution inevitably occurs at any stage of oil production and exerts a negative impact on the environment. Some microorganisms can degrade petroleum hydrocarbons (PHs). Polluted sludge of Rumaila oil field was use to isolate the highly efficient hydrocarbon‐degrading fungal strain. *Aspergillus* sp. RFC‐1 was obtained and its degradation ability for petroleum hydrocarbons was evaluated through surface adsorption, cell uptake, hydrophobicity, surface tension, biosurfactant production, and emulsification activity. In addition, the degradation mechanism was investigated. The results indicated the strain RFC‐1 showed high removal activity for PHs, including biodegradation, adsorption, and emulsifiability. On the day 7 of incubation, the removal efficiencies of crude oil, naphthalene (NAP), phenanthrene (PHE), and pyrene (PYR) reached 60.3%, 97.4%, 84.9%, and 90.7%, respectively. Biodegradation efficiencies of crude oil, NAP, PHE, and PYR were 51.8%, 84.6%, 50.3%, and 55.1%, respectively. Surface adsorption and cell absorption by live mycelial pellets followed a decreasing order: PYR ≥ PHE > NAP > crude oil. Adsorption by heat‐killed mycelial pellets increased within 40 and 10 min for crude oil and PAHs, respectively, and remained constant thereafter. Effects of cell surface hydrophobicity, surface tension, and emulsification index were discussed. Intra‐ and extracellular enzymes of strain RFC‐1 played important roles in PHs degradation. The strain RFC‐1 is a prospective strain for removing PHs from aqueous environments.

## 1 INTRODUCTION

Petroleum hydrocarbons (PHs) are the most important raw materials for industrial chemicals and energy resources used in daily life. However, PHs also act as major environmental pollutants. With the increasing demand for energy, high amounts of PHs from exploitation, refining, storage, and transportation contaminate the environment, especially in the case of large‐scale accidental spills. PHs are categorized into four broad classes of chemical compounds, namely, the aliphatic, aromatic, resin‐based, and asphaltene‐based hydrocarbons. Each group possesses different physicochemical characteristics and susceptibility to degradation (Mishra, Jyot, Kuhad, & Lal, 2001; Sugiura, Ishihara, Shimauchi, & Harayama, 1996). The petroleum type is extremely significant in determining the extent of environmental and socioeconomic damage.

Polycyclic aromatic hydrocarbons (PAHs) are highly toxic carcinogenic, mutagenic, and teratogenic complex compounds and require several years or decades for recovery (Wu et al., 2008; Balachandran, Duraipandiyan, Balakrishna, & Ignacimuthu, 2012). Most PAHs, particularly phenanthrene (PHE) and pyrene (PYR), consist of three or four rings and are medium volatility between gaseous and particle phases in the atmosphere. These molecules can be deposited in surface water and soil where they feature long lifespans (Keyte, Harrison, & Lammel, 2013). Naphthalene (NAP) is a ubiquitous environmental pollutant causing diseases, for example, the respiratory system disease (Ling, Liang, Chung, Lin, & Lin, 2014). Increasing PHs contamination has caused significant environmental concern worldwide. The PHs are released into the environment and may be subjected to oxidation, volatilization, photolysis, and adsorption on sediment and soil particles.

Some microorganisms including bacteria, fungi, and algae, can use PHs as carbon and energy sources, which are converted into carbon dioxide and water or low‐toxic or nontoxic materials (Johnsen, Wick, & Harms, 2005). Biodegradation is anticipated to be an efficient, economic, and environmentally friendly alternative for the removal of petroleum pollutants from contaminated environments and for prevention of damages caused by petroleum spills, compared with other physical and chemical methods, including photolysis, combustion, landfill, ultrasonic decomposition, and addition of dispersants (Margesin & Schinner, 2001; Zhang et al., 2011).

Indigenous microorganisms that can withstand and degrade PHs may exist in water, soil, and sediment contaminated by crude oil and PAHs (Moon, Kahng, Kim, Kukor, & Nam, 2006; Balachandran et al., 2012). Therefore, isolation of highly degrading microorganisms from the natural environment is a feasible and significant technique (Ambrosoli, Petruzzelli, Luis Minati, & Ajmone Marsan, 2005). The abilities to degrade and remove crude oil and PAHs vary with microbial strain and characteristics of pollutants. Some microorganisms can utilize straight, branched, and cyclic alkanes and aromatic hydrocarbons (mainly low‐molecular‐weight PAHs) as sole carbon sources (Luan et al., 2006).

Fungi have certain advantages over bacteria with regard to biodegradation due to their resistance to PHs. Filamentous fungi, *Aspergillus* spp. have been received considerable attention as potential candidates for the degradation of a wide range of PHs from the environment (Ye et al., 2011; Zhang, Xue, Gao, Ma, & Wang, 2016). Two filamentous fungi isolated from a crude oil‐contaminated soil in northeastern Ecuador were efficient for PHs biodegradation (Gooty, 2015). *Aspergillus* and *Rhizopus* sp. collected from automobile workshops in Pudukkottai, Tamilnadu, South India have a good ability to degrade engine oil (Thenmozhi, Arumugam, Nagasathya, Thajuddin, & Paneerselvam, 2013). An *Aspergillus* sp. isolated from Gulf of Mexico has active capability in degrading crude oil (Al‐Nasrawi, 2012). *Aspergillus fumigatu*s from a contaminated environment shows an exceptional ability to degrade anthracene and can metabolize anthracene effectively (Ye et al., 2011). Furthermore, white rot fungi *Irpex lacteus* and *Pleurotus ostreatus* can also degrade PHs satisfactorily (Leonardi et al., 2007). Except for biodegradation, microorganisms also play a significant role in bioadsorption of pollutants in the environment (Stringfellow & Alvarez‐Cohen, 1999). Microorganisms usually secrete enzymes to degrade pollutants. Extracellular enzymes from six *Aspergillus* species, which are isolated from crude‐oil‐contaminated soil, efficiently degrade crude oil (Zhang et al., 2016). Although there are some studies on removal and biodegradation of PHs, a few researchers focused on the surface adsorption and cell absorption of different PHs by microorganisms (Ke, Luo, Wang, Luan, & Tam, 2010; Xu, Bao, Sun, & Li, 2013; Ye et al., 2011). Exploration of the fate of organic pollutants in microbial cell and surface bears importance for understanding the degradation mechanism and transport of pollutants.

This study primarily aimed to evaluate the ability of *Aspergillus* sp. RFC‐1 isolated from petroleum‐contaminated soil for biodegradation and removal of crude oil and two‐, three‐, and four‐ring PAHs. The second objective involved in studying surface adsorption and cell absorption of PHs (crude oil and three PAHs) by mycelial pellets. Cell surface hydrophobicity, biosurfactant production, emulsifiability of mycelial pellets were also investigated. Finally, intra‐ and extracellular enzymes secreted by mycelial pellets were explored.

## 2 MATERIALS AND METHODS

### 2.1 Materials

Crude oil from Rumaila oilfield was obtained from Southern Oil Company (Basra, Iraq). Surface polluted sludge (1–10 cm) was collected Rumaila oilfield (Basra, Iraq). NAP (chemical formula C_10_H_8_) was purchased from Sinopharm Chemical Reagent Co., Ltd. PHE (purity ≥97%; chemical formula C_14_H_10_) and PYR (purity ≥97%; chemical formula C_16_H_10_) were purchased from Aladdin Chemistry Co., Ltd. Crude oil was dissolved in trichloromethane (10 g/L). NAP, PHE, and PYR were dissolved in cyclohexane (2 g/L) as mother liquor.

Mineral salt medium (MSM) contained the following (g/L distilled water): 1.0 K_2_HPO_4_ 3H_2_O, 0.4 KH_2_PO_4_, 0.5 NaCl, 0.1 (NH_4_)_2_SO_4_, 0.2 NaNO_3_, and 0.025 MgSO_4_.7H_2_O. For plate culture, 15 g/L agar was added to liquid MSM. The degradation medium comprised MSM and individual substrates of crude oil, NAP, PHE, and PYR at different concentrations.

### 2.2 Strain isolation and screening

Surface polluted sludge (1–10 cm) was collected from Rumaila oil field. MSM with 1% crude oil was used to feed the microorganisms with oil degradation abilities in soil samples. Five grams of soil samples were cultivated in 250‐ml Erlenmeyer flasks containing 100 ml MSM supplemented with 1% crude oil as a sole source of carbon and energy at 30°C with shaking at 130 r/min for 7 days. Primary inoculums (10 ml) were transferred in fresh MSM with 1% crude oil, incubated for 7 days. The enrichment cultivation was carried out under the same conditions, and repeated four times totally for 4 weeks. The microorganisms detected in the enrichment culture were transferred onto 15 g/L MSM agar plates (sprayed with 1% crude oil). Morphologically separated colonies were picked and purified on fresh potato dextrose agar (PDA) plates at least three times.

After that, a 5 mm mycelial disc from the margin of the actively growing cultures on PDA plate was transferred into 100 ml of potato dextrose broth (PDB) medium in a 250 ml Erlenmeyer flask and incubated at 30°C with shaking at 130 r/min for 3 days as primary inoculums. Then, 10 ml of primary inoculums were inoculated into fresh PDB medium and incubated at the same conditions. After 2 days, mycelial pellets were harvested by centrifugation at 7000 *g* for 5 min at 4°C and washed thrice with sterile MSM. Then, the mycelial pellets were resuspended in MSM, transferred to a sterile Waring blender cup, and homogenized.

For the growth of isolated strains, the optimum conditions were as follows: temperature of 30°C, pH 7, with 250 mg/L crude oil, 50 mg/L NAP, 20 mg/L PHE, and 20 mg/L PYR. To screen oil‐degraded strains, crude oil and PAHs (NAP, PHE, and PYR) were used as sole carbon sources.

### 2.3 Molecular identification of oil‐degraded strain

The isolated fungus was cultured for 3 days in PDB media at 30°C. Mycelia were harvested by centrifugation at 7000 *g* for 5 min at 4°C, frozen in liquid nitrogen, and then ground using a pestle and mortar. Genomic DNA was extracted using cetyltrimethylammonium bromide method described by Doyle (1990). Internal transcribed spacer (ITS) fragments were amplified using two primers: forward primer ITS1F (5ʹ‐TCCGTAGGTGAACCTGCGG‐3ʹ) and reverse primer ITS4R (5ʹ‐TCCTCCGCTTATTGATATGC‐3ʹ). Mixtures of polymerase chain reaction (PCR) reaction contained the following (μl/25 μl): 2× Tsingke Master Mix blue 12.5 (Tsingke Biological Technology Co., Ltd), ITS1F 1, ITS4R 1, template DNA 1, and DNase/RNase‐Free distilled water to obtain a final volume of 25 μl.

Thermocycling for PCR amplifications was performed according to manufacturer's instructions. Initial denaturation was performed at 98°C for 2 min, followed by 35 cycles of denaturation at 98°C for 10 s, annealing at 45°C for 15 s, polymerization at 72°C for 15 s, and final extension at 72°C for 5 min. PCR products were analyzed using 1.5% agarose gel electrophoresis and purified using Axygen DNA Gel Extraction Kit (Axygen, USA) according to manufacturer's instructions. Then, the obtained purified PCR products were directly sequenced by Tsingke (Tsingke Biological Technology, Co., Ltd) using a 3730xl DNA sequencer (ABI, USA). ITS sequences were blasted in National Center for Biotechnology Information. Sequence alignment was performed using ClusterX (version 2.0). Phylogenetic tree was constructed using the neighbor‐joining method, and sequence analyses were conducted using the MEGA software (version 6.0).

### 2.4 Biodegradation of crude oil, NAP, PHE, and PYR

A 10% v/v mycelial suspension of strain RFC‐1 was inoculated into the degradation medium (20 ml containing 250 mg/L crude oil, 50 mg/L NAP, 20 mg/L PHE, or 20 mg/L PYR, respectively, in a 50 ml flask). The flasks were incubated in a shaker at 120 r/min and 30°C. To reduce abiotic losses, the flasks were tightly sealed using aluminum‐foil‐lined Teflon screw caps. The control was prepared in the same conditions without inoculating mycelial pellets.

### 2.5 Crude oil and PAH extraction and analyses

On the day 1, 5, and 7, triplicate flasks were sacrificed, and fungal mycelia were separated from the medium by centrifugation at 7000 *g* for 5 min at 4°C. Crude oil and PAHs in the aqueous supernatant (i.e., those remaining in medium ρ_1_) were extracted with equal volumes (1:1) of chloroform for crude oil and cyclohexane for PAHs using the liquid‐liquid extraction method (Xu et al., 2013). The total crude oil was determined spectrophotometrically following the method of Zhao, Wang, Ye, Borthwick, and Ni (2006) using the Alpha‐1860 Ultraviolet Spectrophotometer (Shanghai Lab‐Spectrum Instruments Co. Ltd., Shanghai, China) at a wavelength of 245.5 nm. The oils and oil products have certain characteristic absorbance at UV range, which the heavy oil corresponds to absorbance wavelength of 250–260 nm, whereas the clean oil corresponds to the absorbance at 215–230 nm (Lagesson, Lagesson‐Andrasko, Andrasko, & Baco, 2000).

For PAHs, the extracted organic layer was pooled, dried using anhydrous sodium sulfate, and evaporated using a vacuum rotary evaporator at room temperature. The extracted samples were finally dissolved in 1 ml of cyclohexane and kept at 4°C for further analysis by gas chromatography (GC).

The residual PHs in mycelial pellets were extracted according to the method of Chen and Wang (2011). The mycelial pellets were suspended in 10 ml of Tris‐HCl (pH 7.5, 0.01 mol/L) and ultrasonicated in an ice bath with 3 s pulse and 7 s pause for 6 min. This fraction was experimentally defined as crude oil and PAHs adsorbed on mycelial pellet surfaces and absorbed by mycelial pellets (ρ_mρ_), (Chen & Wang, 2011; Xu et al., 2013). Residual concentrations of PHs in the medium and mycelial pellets were determined.

Quantification of removal and biodegradation of PHs used an external standard method using a MIDI Sherlock GC (Agilent, USA) equipped with a flame ionization detector. PHs were separated using a 19091z‐433 PH‐1 capillary column (30 m × 0.25 mm × 0.25 μm) under the following conditions: nitrogen was used as carrier gas at a flow rate of 42.1 ml/min; flow rates of hydrogen gas and air measured 30.0 and 400.0 ml/min, respectively; initial and final oven temperatures were 35 and 280°C (at a rate of 5°C/min), respectively; injector and detector temperatures were both set at 280°C; temperature program rate was 8°C/min, and final hold time for the oven was 5 min; injection volume totaled 5 μl with a split ratio of 10; and total run time spanned 35.6 min.

To estimate the removal (based on residual amounts in the degradation medium) and biodegradation efficiencies of PHs in the degradation media, removal rate (*R*) and degradation rate (*D*) were, respectively, calculated using the following equations:

(1) $$R=\left(\rho_{0}-\rho_{1}\right)/\rho_{0}\times 100\%$$

(2) $$D=\left(\rho_{0}-\rho_{1}-\rho_{\mathrm{mp}}\right)/\rho_{0}\times 100\%$$

where ρ_0_ refers to the initial concentration of PHs (mg/L); ρ_1_ is the remaining PHs concentration at different incubation periods (mg/L); and ρ_mp_ indicates the concentration of PHs adsorbed onto the cell surface and absorbed by mycelial pellets (mg/L).

The removal of crude oil, NAP, PHE, and PYR in MSM were measured, and specific degradation rate was expressed by the following formula:

(3) $$dx/x_{0}\cdot dt$$

where d*x* denotes the change in substrate concentration; *x*_0_ stands for the initial substrate concentration; and d*t* is the time interval.

### 2.6 Degradation kinetics

Biodegradation kinetics can help us to further comprehend the mechanism of fungal degradation. As degradation of crude oil, NAP, PHE, and PYR fits the first‐order reaction kinetics, it can be calculated using the following formula:

(4) $$-\text{In}C_{t}/C_{0}=Kt$$

where *C*_*t*_ refers to the residual PHs concentration at any time (mg/L), *C*_*0*_ denotes the initial PHs concentration (mg/L), *K* indicates the observed first‐order reaction rate constant for PHs biodegradation (day^−1^), and *t* is time (day).

Half‐life period of PHs was also calculated using the following formula:

(5) $$t_{1/2}=\text{In}\;2/k$$

where *k* represents the biodegradation rate constant (day^−1^).

### 2.7 Surface adsorption and absorption

#### 2.7.1 Surface adsorption and cell absorption of PHs by live mycelial pellets

A 10% v/v homogenized mycelial suspension was inoculated into MSM with 100 mg/L crude oil and 20 mg/L NAP, PHE, or PYR. After sequential incubation periods of 1, 5, 10, 20, 30, 40, 60, 90, 180, 360, 720, and 1440 min, mycelia were harvested by centrifugation at 7000 *g* for 5 min at 4°C. Mycelial pellets were washed with sterile MSM, twice with a solvent mixture (ethanol/butanol/chloroform = 10/10/1 by volume) to remove cell surface‐associated hydrocarbons (Chen & Wang, 2011; Kim, Foght, & Gray, 2002), and twice again with sterile MSM. The extracted hydrocarbons were used to quantify surface‐adsorbed hydrocarbons by mycelial pellets. Crude oil concentration in the extracted hydrocarbons was determined at an optical density of 245.5 nm (OD_245.5_), whereas concentrations of NAP, PHE, and PYR were determined using GC. All experiments were performed in duplicate.

The mycelial pellets used surface adsorption were suspended in 10 ml of Tris‐HCl (0.01 mol/L, pH 7.5) and broken up ultrasonically in an ice bath with 3 s pulse and 7 s break 60 times. The samples were centrifuged at 7000 *g* at 4°C for 10 min, and PH fractions in the supernatant were experimentally defined as mycelial‐pellet‐absorbed PH (Chen & Wang, 2011; Xu et al., 2013). All experiments were performed in duplicate.

#### 2.7.2 Surface adsorption of PHs by heat‐killed mycelial pellets

The homogenized mycelial suspension was autoclaved at 121°C for 20 min. The heat‐killed mycelial suspension (10%, v/v) was inoculated into MSM with 100 mg/L crude oil and 20 mg/L NAP, PHE, or PYR. After sequential incubation periods of 1, 10, 30, 40, 50, 60, and 90 min at 30°C with shaking at 120 r/min, mycelia were harvested by centrifugation at 7000 *g* for 5 min at 4°C. Concentrations of crude oil, NAP, PHE, and PYR in individual supernatants were determined. All experiments were performed in duplicate.

#### 2.7.3 Effects of pH and temperature on surface adsorption of PHs by live mycelial pellets

Effects of pH and temperature on surface adsorption of PHs by live mycelial pellets were investigated over a pH range of 2.0–8.0 at 30°C and at an incubation temperature range of 10–40°C at pH 7.0. Adsorption experimental procedures were the same as in subsection 3.8. Adsorption time was 30 min. All experiments were performed in duplicate.

### 2.8 Measurement of cell surface hydrophobicity of mycelial pellets and liquid surface tension

Fungal adhesion to hydrocarbons (FATH) was used to test cell surface hydrophobicity of the strain RFC‐1 with PHs, according to the method developed by Pruthi and Cameotra (1997). FATH represents the hydrophobic potential of fungi, that is, the percentage of adherence of fungal cells to hydrophobic substrates. In this assay, mycelial suspension was added into a screw cap test tube (10 ml) containing MSM with 20 mg/L of each substrate (crude oil, NAP, PHE, and PYR) and cultivated at 30°C with shaking at 120 r/min for 1, 3, 5, and 7 days. Subsequently, the suspension was vortex‐shaken at high speed for 2 min. The media were kept at 30°C for 1 hr to stratify the PH phases and aqueous phase completely. All initial and final absorbances (*A*, less than 1) of mycelial suspensions in the aqueous phase were measured at 600 nm (Chen & Wang, 2011; Xu et al., 2013). The percentage of cell surface hydrophobicity was calculated as follows:

(6) $$\text{FATH}=\left[1-\left(A_{\mathrm{bs}1}/A_{\mathrm{bs}0}\right)\right]\times 100\%$$

where FATH represents the hydrophobicity percentage of the fungus, and *A*_bs0_ and *A*_bs1_ correspond to initial and final absorbances at 600 nm, respectively.

Surface tension of aqueous supernatants was determined using Force Tensiometer‐K100 (Hamburg, Germany). Surface tension was tested in MSM containing 20 mg/L crude oil, NAP, PHE, and PYR inoculated with the mycelial suspension. Surface tensions of the samples were measured on the day 1, 3, 5, and 7. All experiments were performed in duplicate.

### 2.9 Screening for biosurfactant production and emulsification activity

Two distinct methods were applied to estimate biosurfactant production. Surfactant was detected from the cell‐free supernatant. The supernatant of the strain RFC‐1 was cultivated in MSM containing 20 mg/L individual crude oil, NAP, PHE, and PYR at 30°C for 7 days and was recovered by centrifugation at 7000 *g* for 5 min and filtered through a 0.22 μm pore‐size filter to obtain a cell‐free supernatant.

The first biosurfactant screening technique was the drop collapse test, in which 7 μl of mineral oil was added to each well of the 96‐well microtiter plate lid. The lid was equilibrated for 24 hr at room temperature. The cell‐free supernatant (20 μl) was then added to the oil surface of a microtiter well (Mahjoubi et al., 2013). Sterile distilled water and Tween‐80 were used as negative and positive controls, respectively. After 1 min, drop shape on oil surface was investigated. Flat and round drops were scored as positive and negative, respectively, in the biosurfactant‐containing supernatant.

The second method was oil displacement test, in which 10 μl of crude oil was added to the surface of 40 ml sterile distilled water into a 90 mm petri dish to form a layer on the surface. Then, 10 μl of cell‐free supernatant was gently placed on the crude oil surface (Morikawa, Hirata, & Imanaka, 2000). Immediate manifestation of a clear zone indicated biosurfactant activity. The diameter of the clear oil‐displaced circle was measured.

Emulsification activity (*E*_24_) was measured using the combination of 2 ml cell‐free supernatant with 2 ml each of crude oil, NAP, PHE, and PYR into a screw cap test tube. The mixture was stood for 24 hr and then vortexed at high speed for 2 min. Emulsification activity was then observed (Bento, de Oliveira Camargo, Okeke, & Frankenberger, 2005). The following equation was used to determine emulsification activity (*E*_24_):

(7) $$E_{24}=HE_{L}/TH_{S}\times 100\%$$

where *E*_24_ indicates the percentage of emulsification activity (%), *HE*_L_ is the height of the emulsified layer (mm), and *TH*_S_ refers to the total height of solution column (mm). All experiments were performed in duplicate.

### 2.10 Ability of intra‐ and extracellular enzymes in biodegradation of PHs

To gain insight into the degradation mechanism, intra‐ and extracellular enzymes were evaluated. Osmotic shock method was used to assemble the enzymes in different cell parts (Meng, Li, Bao, & Sun, 2017; Yoo, Park, Kim, Choi, & Yang, 2012). 10% homogenized mycelial suspension was inoculated into the degradation medium containing 20 mg/L crude oil, NAP, PHE, or PYR. After 3 days of shaking at 30°C and 120 r/min, the culture medium was centrifuged at 10,000 *g* for 10 min at 4°C.

The supernatant (S_1_) was filtered using a 0.22 μm pore‐size filter to remove the mycelia. Pellet P_1_ was washed twice with cold distilled water, resuspended in 10 ml Tris–HCl (0.01 mol/L, pH 8), centrifuged at 10,000 *g* for 10 min at 4°C, and filtered using a 0.22 μm membrane filter (S_2_). Pellet P_2_ was mixed with 10 ml sucrose liquid (25%). After 10 min of shaking at 30°C at 120 r/min, the mixture was centrifuged at 10,000 *g* for 10 min at 4°C and also filtered using a 0.22 μm membrane filter (S_3_). Pellet P_3_ was resuspended in 10 ml Tris–HCl (0.01 mol/L, pH 7.5). Cell rupture was performed in an ice bath, followed by ultrasonication for 6 min (3 s pulse and 7 s break), centrifugation at 15,000 *g* for 20 min at 4°C, and filtration using a 0.22 μm membrane filter (S_4_). Filtrates S_1_, S_2_, S_3_, and S_4_ were collected and kept at 4°C (Chen & Wang, 2011; Xu et al., 2013; Ye et al., 2011).

Extracellular enzyme (mixed S1, S2, and S3) and intracellular enzyme (S_4_) (10%, v/v) was inoculated into 20 mg/L of each substrate (crude oil, NAP, PHE, and PYR) degradation media and cultivated at 30°C and 120 r/min for 1, 5, and 7 days, respectively. All experiments were performed in duplicate. The control experiment was performed under the same conditions.

### 2.11 Statistical analysis

All experiments were performed in independent duplicate. Mean and standard deviations of the three experiments were calculated. Statistical analysis was performed using OriginLab 2017.

## 3 RESULTS AND DISCUSSION

### 3.1 Molecular identification of the strain RFC‐1

ITS sequence is usually used to identify isolated fungi. ITS region of the oil‐degraded RFC‐1 strain was sequenced and submitted to GenBank (accession no. KY328441). This strain belongs to the genus *Aspergillus* (Figure S1).

### 3.2 Surface adsorption of PHs by live mycelial pellets

**Figure 1:**
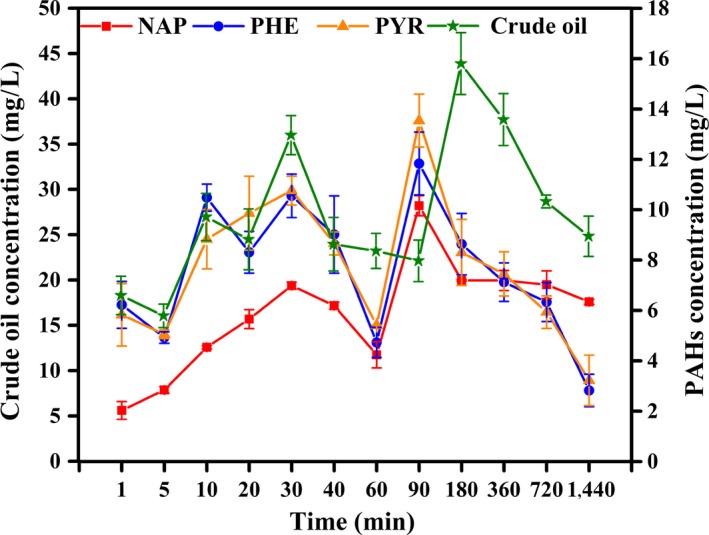
Surface adsorption of PHs by live mycelia pellets

Figure 1 shows the time courses of crude oil, NAP, PHE, and PYR adsorbed by live mycelial pellets from 1 min to 1440 min. Trends of surface adsorption of crude oil, NAP, PHE, and PYR gradually increased as a whole but occasionally decreased. This result was partly attributed to reversible biosorption‐desorption (Chen, Wang, & Hu, 2010; Ding, Wang, Shen, & Chen, 2010). The highest percentage of surface adsorption totaled 43.8% at 180 min for crude oil, 50.5%, 59.0%, and 67.2% at 90 min for NAP, PHE, and PYR, respectively. This showed PAHs were more easily adsorbed by mycelial pellets than crude oil. PHE and PYR were also adsorbed more easily by live mycelial pellets than NAP. This result was consistent with that of Xu et al. (2013) using microbial consortium. This result also indicated that the live mycelial pellets adsorbed PHs selectively.

The medium contained significantly high initial PHs concentration, causing the substrates to move from high‐ to low‐concentration cell surface. Thus, this process may not be related to biodegradation during a brief period. Adsorption efficiency of an adsorbent depends on its ability to adsorb a particular adsorbate. Surface area of an adsorbent also plays a significant role in adsorption of PHs from its aqueous solution (Bairagi, Khan, Ray, & Guha, 2011).

### 3.3 Adsorption of PHs by heat‐killed mycelial pellets

**Figure 2:**
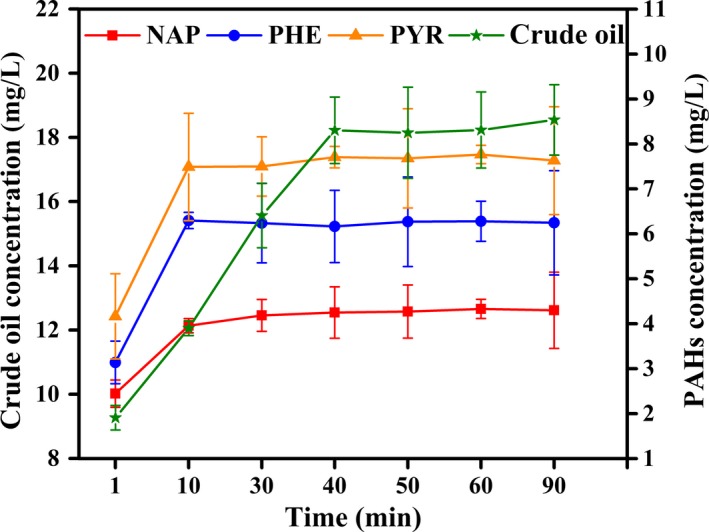
Adsorption of PHs by heat‐killed mycelial pellets

PAHs adsorption by dead fungal biomass increased rapidly from 1 min to 10 min, whereas the increase in crude oil adsorption lasted for 40 min (Figure 2). Maximum adsorption percentages of NAP, PHE, and PYR by dead fungal biomass were 21.5%, 31.9%, and 38.0%, respectively, and followed the order of PYR > PHE > NAP, with PYR manifesting the highest hydrophobicity. The results were similar to those of live mycelia adsorption, but adsorption percentage was lower than that of live mycelia. These findings also indicated the selective absorption of dead fungal mycelia in this process. Adsorption of crude oil by dead fungal mycelia gradually increased with time and reached a maximum percentage of 18.2% within 40 min.

Thereafter, adsorption remained at a stable rate probably because of the complex mixture of crude oil comprising *n*‐alkanes and aromatics, resins, and asphaltenes, some of which were slowly adsorbed on the surface of dead cells. Chen et al. (2010) observed that bioadsorption of heat‐killed cells was related to carbon‐normalized partition coefficients. Ding, Chen, and Zhu (2013) explored the respective partition coefficients of PHE and PYR and pointed out that removal of PAHs by heat‐killed bodies of *Phanerochaete chrysosporium* was only attributed to the mechanism of bioadsorption. These findings indicated the rapid adsorption of PHs by heat‐killed mycelial pellets. Adsorption content was constant for the three PAHs and crude oil. Ding et al. (2010) reported that the biosorption mechanism of nonpolar organic pollutants by dead biomass is partition.

Yuan, Tong, Zhao, and Jia (2010) showed that most of PAH sorption occurred during the first 30 min using petroleum‐coke‐derived porous carbon as adsorbent to adsorb PAHs from water and the adsorption percentages of NAP, PHE, and PYR amounted 99.1%, 95.2%, and 93.4%, respectively, which was higher than the maximum surface and cell bioadsorption content by live and dead microorganism at experimental time (comparing with Figures 1 and 2).

Thus, efficiency of physicochemical adsorption was significantly higher than that of bioadsorption. Given the high cost of excavation and transportation of large quantities of contaminated materials for ex situ treatment, various traditional engineering‐based physicochemical decontamination techniques present limitations (Farhadian, Vachelard, Duchez, & Larroche, 2008). Biological removal and biodegradation technologies are consistently expected to be an efficient, economic, and environmentally friendly alternative for pollutant cleanups (Zhang et al., 2011).

### 3.4 Effect of pH and temperature on adsorption of PHs by live mycelial pellets

**Figure 3:**
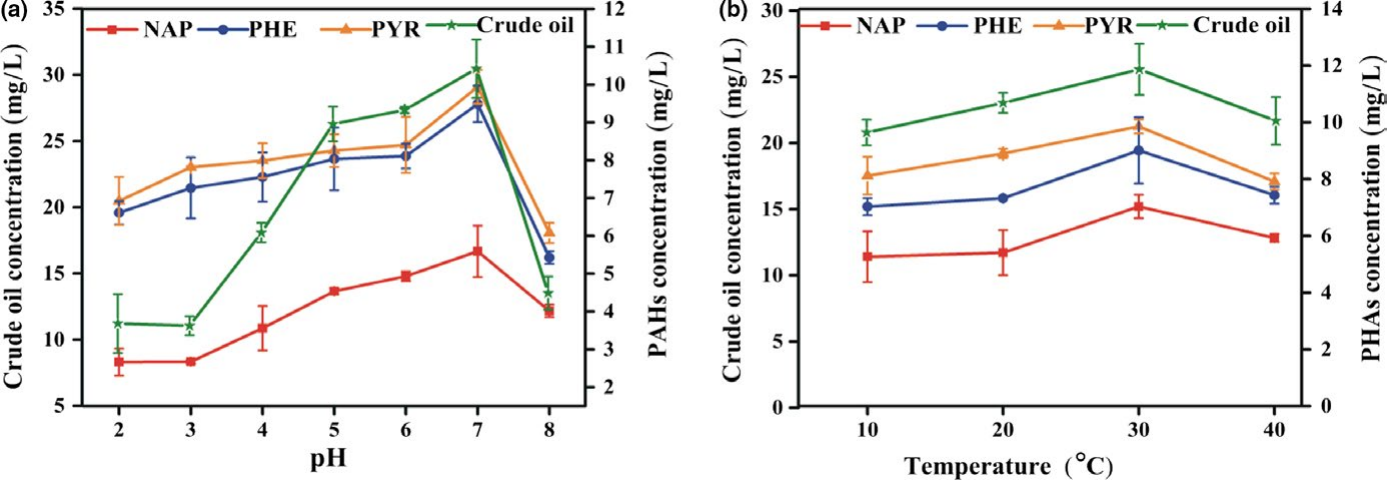
Effect of pH (a) and temperature (b) on surface adsorption of PHs by live mycelial pellets incubated for 30 min

The adsorption of crude oil and the three PAHs increased with pH values ranging from 3.0 to 7.0 and decreased at pH 8.0. The highest adsorption of PHs by mycelial pellets of the strain RFC‐1 was occurred at pH 6–7 and significantly decreased at pH 8.0 (Figure 3a). These findings suggest that increasing hydrogenions inhibit the adsorption of PHs to cation‐binding sites (Jalali, Ghafourian, Asef, Davarpanah, & Sepehr, 2002). Lower PHs adsorption at acid pH may be due to the competition for cation‐binding sites between hydrogen (H^+^) and hydronium (H_3_O^+^) ions in the solution (Jalali et al., 2002) or reduced solubility of PHs and/or precipitation of ions (Zhou & Kiff, 1991).

The effect of temperature (10–40°C) on adsorption of crude oil, NAP, PHE, and PYR by the strain RFC‐1 was determined at pH 7.0 (Figure 3b). Adsorption increased with temperature ranging from 10 to 40°C, and optimal temperature for adsorption was 30°C. The increase in adsorption with specific temperature range was possibly due to the increased energy efficiency in the system, facilitating the attachment of substrates on the cell surface, whereas the decline with further rise in temperature possibly resulted from the distortion of some cell sites available for substrate surface adsorption or adsorption that may occur with high temperatures (Al‐Asheh & Duvnjak, 1995).

### 3.5 Cell absorption of PHs by live mycelial pellets

**Figure 4:**
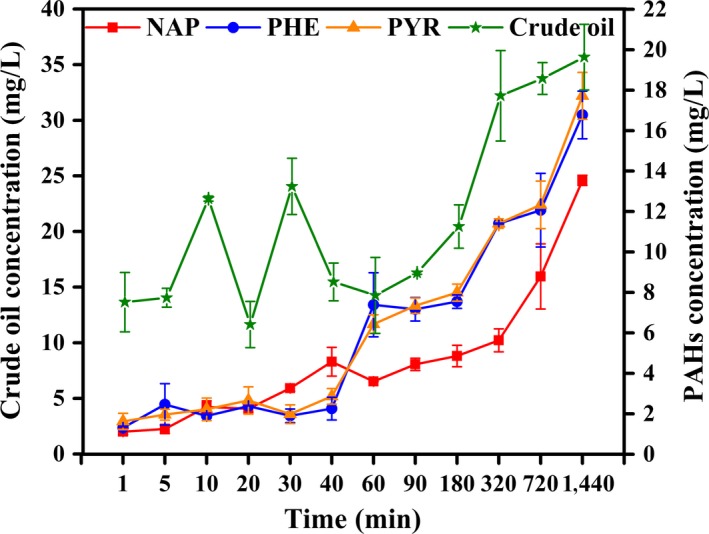
Cell absorption of PHs by live mycelia pellets

Mycelial pellets exhibited a remarkable capacity for absorbing crude oil, NAP, PHE, and PYR. After 60 min, absorption capacity increased with time (Figure 4). The highest absorption percentages of crude oil, NAP, PHE, and PYR by mycelial pellets amounted to 35.7%, 67.7%, 83.9%, and 88.9% at 1440 min, respectively. These values were higher than their surface adsorption percentages. Absorption and surface adsorption of NAP by mycelial pellets were lower than those of PHE and PYR. This absorption process may be associated with biodegradation.

The accumulation in fungal biomass suggested that the mycelial pellets entrapped PHs as they occurred in the aqueous phase. Three possible mechanisms of hydrocarbon absorption were proposed (Beal & Betts, 2000). The first was direct absorption of hydrocarbons dissolved in the aqueous phase by microbial cells. The direct absorption is a naturally limited process because of the low solubility of most hydrocarbons. In other words, this mechanism is assumed to operate for the uptake of small chain types (Hommel, 1990). The second possible mechanism involves the microbial cells absorbing the hydrocarbon droplets, which are melted or melted‐like and smaller than cells. The third was that hydrocarbons larger than cells are transported inside the cells by direct contact. In this hypothesis, microbial cells directly make contact with hydrocarbons that are much larger than cells and then absorb hydrocarbons. The substrate absorption is thought to occur through active transport or diffusion.

As PHs, particularly PAHs, feature strong hydrophobicity, mycelial pellets may absorb PHs by the second or the third mechanism. Studies have proven that some fungi take up and store PAHs in lipid vesicles although the extent of absorption and the mechanisms by which PAHs cross the cell wall remain unknown (Wu et al., 2009). Results of surface adsorption and cell absorption of PHs and the trend of transportation and absorption of substrates suggest that transport pathways of crude oil and PAHs by the mycelial pellets probably differed. For crude oil, surface adsorption and cell absorption by mycelial pellets slightly increased, whereas those for the three PAHs increased markedly. Substrate constituents and selective absorption of the cells were possibly related to these processes.

### 3.6 Biosurfactant, emulsifiability, and hydrophobicity of mycelial pellets and surface tension in the degradation medium

Two methods were used to detect biosurfactant production, namely, the drop collapse test and oil spreading test. The first method showed positive and negative results for biosurfactant production (data not shown). This method cannot detect low levels of biosurfactant production (Youssef et al., 2004). This technique is easily used to examine a large number of samples, but it has not been correlated with surface tension reduction to confirm its reliability (Bodour, Drees, & Maier, 2003).

All samples were positive in the oil spreading test (Table S1), which can detect biosurfactant production in low levels (Mahjoubi et al., 2013). The diameter of clear zone was proportional to the concentration of biosurfactant production. Biosurfactant production of crude oil, NAP, PHE, and PYR by the mycelial pellets followed the order crude oil ≥ PYR > PHE ≥ NAP. To detect biosurfactants at low levels, cultures should be initially screened using the drop collapse method, followed by negative culture screening by oil spreading test.

Emulsification potential is another index for screening the efficiency of biosurfactant‐producing microorganisms. The highest emulsification activities of crude oil, NAP, PHE, and PYR by the mycelial pellets reached 35.8%, 30.1%, 32.5%, and 37.0%, respectively, and followed the order PYR > crude oil > PHE > NAP. These results agreed with the relative correlation between production of biosurfactant and emulsification (Mahjoubi et al., 2013).

Hydrophobicities of mycelial pellets cultivated in the crude oil, NAP, PHE, and PYR medium reached more than 70% on the first day and 54% at 7 days, implying the notable emulsifying efficiency and high degradation activity of mycelial pellets (Hassanshahian, Tebyanian, & Cappello, 2012). Some researchers have reported the relationship between cell surface hydrophobicity and degradation of crude oil by microorganisms (Mehdi & Giti, 2008). Hydrophobicity of mycelial pellets reduced gradually during degradation. This decrease was probably attributed to the gradual depletion of the carbon source caused by substrate degradation by mycelial pellets, thus weakening the emulsifiability of mycelial pellets for PHs. In addition, hydrophobicity may be associated with substrate concentration and toxicity (Zhang et al., 2010). However, the decrease in hydrophobicity is probably related with the production of biosurfactant, which can alter the hydrophobicity of the surface, and in turn affect the adhesion of microbes over the surface (Vijayakumar & Saravanan, 2015).

**Figure 5:**
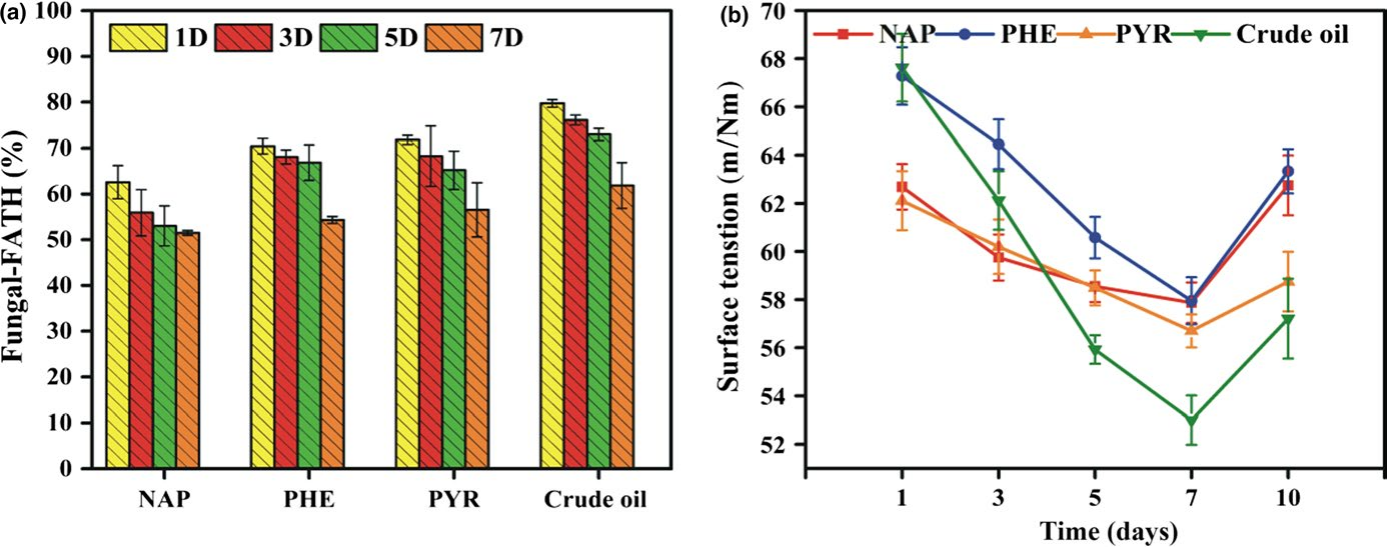
Hydrophobicity of mycelial pellet (a), and surface tension in biodegradation medium (b)

Surface tension in crude oil dropped dramatically, compared with that in PAHs within 5 days (Figure 5b). Biosurfactants produced by the mycelial pellets played significant roles in limiting the surface tension of supernatant in crude oil, compared with in PAHs. Results also illustrated that mycelial pellets in crude oil exhibited high‐biosurfactant production, which dramatically decreased surface tension, coinciding with the results of Hassanshahian et al. (2012), who reported the correlation of biosurfactant production, emulsification activity (*E*_24_), and reduced surface tension with increasing growth rate on hydrocarbons. However, after 5 days, surface tension in the crude oil and PAHs increased, and this condition most possibly resulted from microorganism metabolites and degradation products of PHs.

The above results indicated that the strain RFC‐1 served as biosurfactant producer and exhibited an acceptable level of emulsification activity on different hydrocarbon substrates. Biosurfactants can act as emulsifying agents by decreasing surface tension. Biosurfactants improve hydrocarbon biodegradation through two mechanisms. The first mechanism promotes emulsification of hydrophobic compounds to mycelial pellet structures. The second mechanism induces high cell surface hydrophobicity, thus increasing the direct physical contact between cells and poor water‐soluble substrates (Vasileva‐Tonkova, Galabova, Stoimenova, & Lalchev, 2008).

Zhang et al. (2011) reported that biosurfactant producers play a critical role in modifying cell surface hydrophobicity. Batista, Mounteer, Amorim, and Totola (2006) observed that the strains isolated from petroleum‐contaminated sites produced biosurfactants and reduced the surface tension below 40 mN/m. Mishra, Singh, and Pande (2014) also reported that the *Pseudomonas aeruginosa* PSA5 decreased surface tension from 70 mN/m to 40 mN/m in MSM with fluoranthene. Biosurfactants with high surface activity and environmental compatibility are generally used for rapid oil‐contaminated soil degradation (Hassanshahian et al., 2012).

### 3.7 Removal and biodegradation of PHs by live mycelial pellets

**Figure 6:**
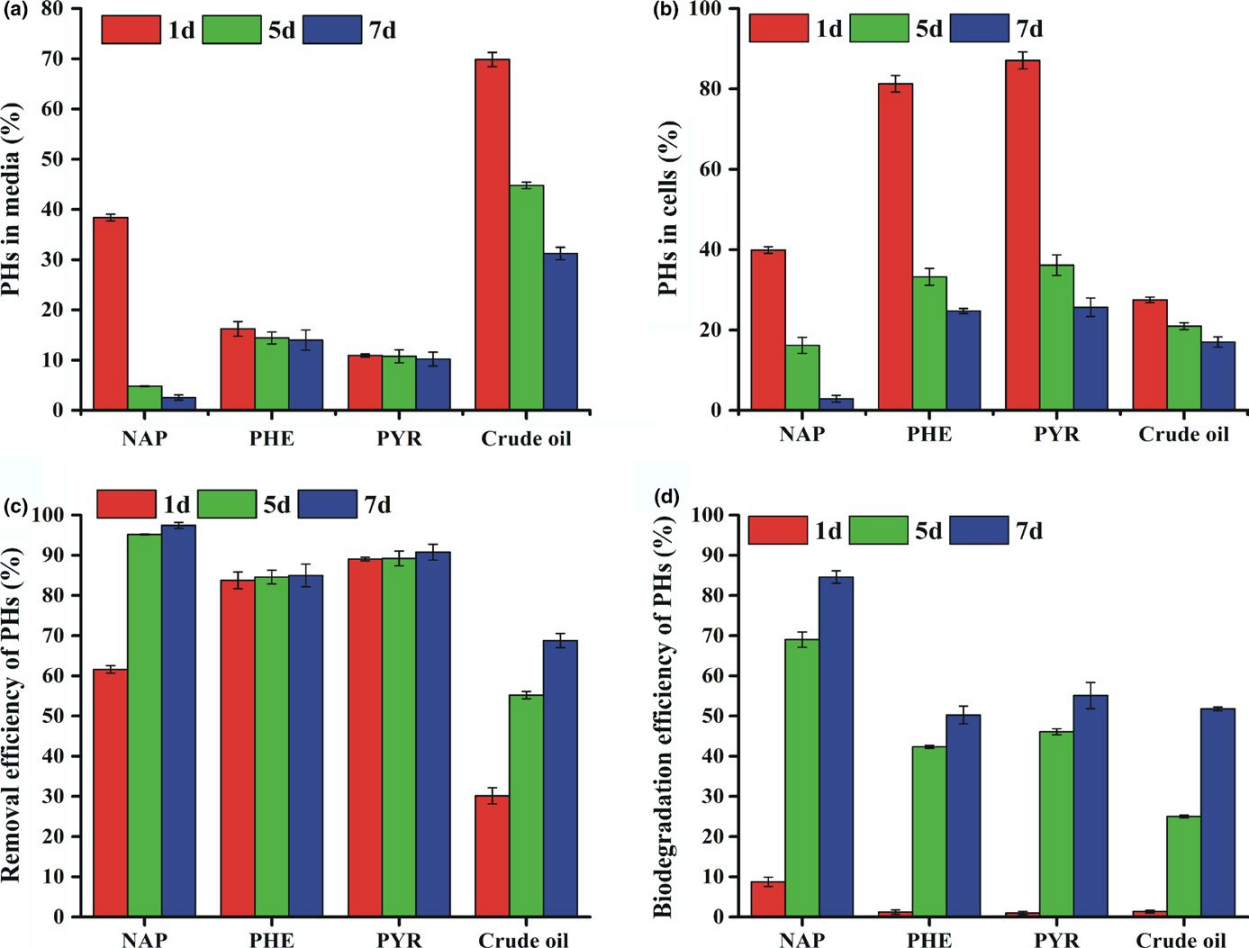
Percentage of crude oil, NAP, PHE, and PYR remained in medium (a); accumulated in the cells (b); removed (c) and biodegraded (d) by the strain RFC‐1 cultivated in degradation media for 1, 5, and 7 days, at pH 7.0, 30°C, and 120 r/min. The initial concentration of crude oil, NAP, PHE, and PYR was 250, 50, 20, and 20 mg/L, respectively

PHs removal includes not only biodegradation but also transport and accumulation. In this study, the amounts of PHs removal, biodegradation, and bioaccumulation (adsorbed onto cell surfaces and absorbed into cells) were examined individually to illustrate the relationships with the biological processes. The amount of PHs remained in the degradation medium inoculated with mycelial suspension decreased substantially on day 1 (Figure 6a). PHE and PYR decreased rapidly, but crude oil and NAP decreased slowly, especially after 1‐day exposure.

To reveal the contributions of metabolism‐independent bioaccumulation to PHs removal, PHs accumulation by live mycelial pellets was investigated (Figure 6b). On day 1 of incubation, bioaccumulations of PHE and PYR were rapid, whereas those of crude oil and NAP were slow. These results may be related to the selective biosorption of cells and cell wall composition. The magnitude of bioaccumulation of organic toxicants in microbes is species‐specific (Lei, Hu, Wong, & Tam, 2007). Within 1 day of incubation, the mycelial pellets adsorbed more than 27.5%, 39.9%, 81.3%, and 87.0% of crude oil, NAP, PHE, and PYR, respectively, from the degradation medium (Figure 6b).

These findings indicated that PHs bioaccumulation was primarily dependent on the physicochemical interactions between PHs and mycelial pellets. Accumulation onto the mycelial pellets reached high levels on day 1 for all four hydrocarbons and continuously decreased with time due to biodegradation (Figure 6b). With increasing incubation time, biodegradation gradually increased, and stored PHs in the fungal bodies correspondingly decreased.

The relationship between biosorption and biodegradation was explained by Stringfellow and Alvarez‐Cohen (1999). Removal of crude oil, NAP, PHE, and PYR within 1 day was related to physicochemical adsorption, and their efficiencies reached 30.2%, 61.7%, 83.8%, and 89.1%, respectively. The main contributors to removal of PHs on the day 1 were physicochemical adsorption and bioaccumulation (Figure 6c). These results indicated that primary PH removal was a passive biosorption, and bioaccumulation served as an important process for removal of PYR and PHE, followed by NAP and crude oil from degradation medium in short periods.

Similar results have been reported by Chen et al. (2010), who revealed that removal efficiencies of PHE and PYR using a consortium of white‐rot fungi reached 80% and 90% in liquid media, respectively. Removal of organic pollutants using microbial bodies commonly occurs in the environment. Microbial remediation is one of the major potential technologies for reducing PHs (Haritash & Kaushik, 2009).

After 1 day of incubation (Figure 6c), most PHs were removed from the degradation medium due to adsorption/absorption rather than biodegradation. Degradation efficiency of PHs increased progressively with incubation time. After rapid adsorption and absorption, PHs were gradually degraded by mycelial pellets along with 51.8%, 84.6%, 50.3%, and 55.1% of crude oil, NAP, PHE, and PYR, respectively (Figure 6d). These results suggest that the strain RFC‐1 can degrade PHs, and the mechanisms possibly involve in two processes: a rapid initial passive physiochemical adsorption followed by absorption, accumulation, and degradation by mycelial pellets.

Degradation and bioaccumulation of PHs occurred concurrently upon their removal from the solution. Removal efficiencies of crude oil, NAP, PHE, and PYR achieved 60.3%, 97.4%, 84.9%, and 90.7%, respectively on the day 7. Residual concentrations of PHs in the control without mycelial pellets showed no significant changes within 7 days. However, 3%, 5%, 2.3%, and 2% reductions were observed in crude oil, NAP, PHE, and RYR, respectively, suggesting that abiotic loss was negligible. Specific degradation rates of crude oil, NAP, PHE, and PYR by the strain RFC‐1 were 0.074, 0.120, 0.071, and 0.078 day^−1^, respectively. However, in the control without any mycelial pellets, specific degradation rates totaled 0.004, 0.007, 0.003, and 0.002 day^−1^ for crude oil, NAP, PHE, and PYR, respectively. When degradation rate constant and half‐life periods of crude oil, NAP, PHE, and PYR were determined following the first‐order kinetics model equation, rate constant was the highest in NAP (1.856 day^−1^, *t*_1/2_ = 0.373) followed by PYR (0.478 day^−1^, *t*_1/2 _= 1.450), PHE (0.422 day^−1^, *t*_1/2_ = 1.64), and crude oil (0.389 day^−1^, *t*_1/2_ = 1.781) in a decreasing order. After 7 days of incubation, no significant difference in rate constant and half‐life periods was identified between PYR, PHE, and crude oil during degradation by the strain RFC‐1. Thus, half‐life period was low with high degradation constant in NAP than in either crude oil, PHE, and PYR.

In this study, degradation efficiency was close to some reports, in which biodegradation rates of crude oil and NAP reached 48% and 80%, respectively (Shen et al., 2015). Bacteria and yeast isolated from tropical soils degrade 52% and 69% of crude oil (Ijah, 1998). *Pestalotiopsis* sp. NG007 can degrade 48%–96% of all the PHs tested (Yanto & Tachibana, 2013). *Penicillium commune* and *A. niger* can degrade 48% and 54% of crude oil, respectively (El Hanafy et al., 2015). It was also reported that two crude oil‐degrading fungi isolated from the Rumaila oilfield, *Penicillium* sp. RMA1 and RMA2 could grow in media containing crude oil and further degrade (57% and 55%, respectively), illustrating that those two fungi could metabolize crude oil efficiently (Al‐Hawash et al., 2017).

Degradation efficiency of crude oil was lower than that of PAHs, which may be attributed to the complex mixture of crude oil consisting of insoluble compounds, such as different chain‐length *n*‐alkanes, resin, and asphalt with poor scattering in water. Some isoalkanes are spared as long as *n*‐alkanes, which are available as substrates, whereas some aromatics are metabolized only in the presence of easily utilized PHs (Okoh, 2006). Among the three PAHs tested in the current study, NAP exhibited the highest degradation efficiency, followed by PYR, and PHE yielding the lowest degradation efficiency. These results were consistent with other research, revealing that one ring is degraded at a time in polycyclic aromatics using a similar method. *Bacillus fusiformis* exhibited a high NAP degradation percentage of 99.1% (Lin, Gan, & Chen, 2010). Biodegradability decreases with growing number of rings and degree of condensation (Atlas & Bartha, 1986; Shen et al., 2015). Degradation efficiency of PYR by the strain RFC‐1 was a little higher than that of PHE. This finding indicates that the strain RFC‐1 more effectively removes high‐molecular‐weight PAHs. Fluoranthene and PYR are degraded more easily by *Selenastrum capricornutum* than PHE (Chan, Luan, Wong, & Tam, 2006). *Coniothyrium* sp. and *Fusarium* sp. isolated from PAH‐contaminated soil preferentially degrades high‐molecular‐weight PAHs with five and six rings over low‐molecular‐weight PAHs (Potin, Rafin, & Veignie, 2004). The degradation order of the three PHs by the strain RFC‐1 was consistent with the results of Xu et al. (2013). These results showed that the strain RFC‐1 could effectively remove PHs via biodegradation and bioaccumulation.

### 3.8 Roles of intra‐ and extracellular enzymes in biodegradation of PHs

**Figure 7:**
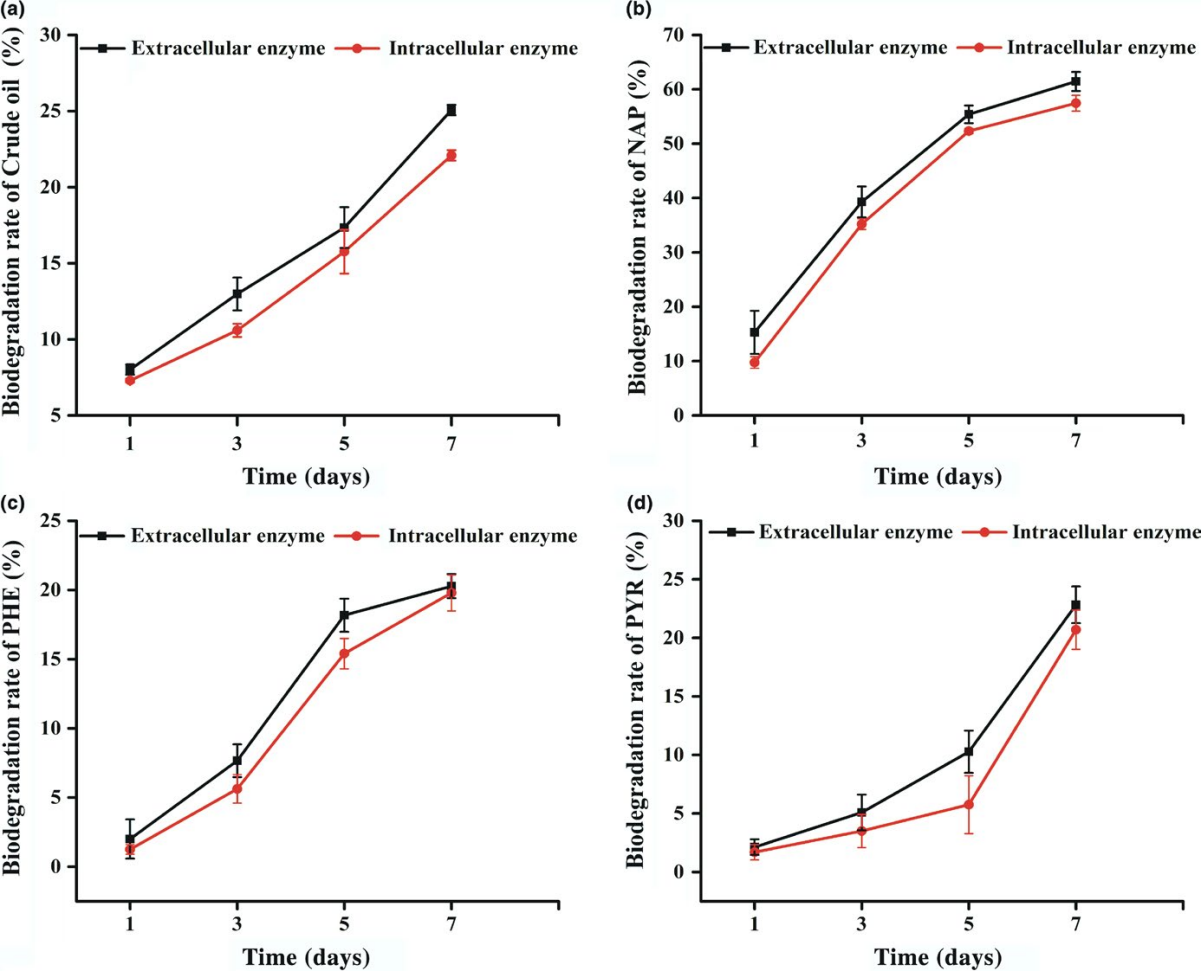
Degradation process of NAP by intracellular and extracellular (a), Degradation process of PHE by intracellular and extracellular (b), Degradation process of PYR by intracellular and extracellular (c) Degradation process of crude oil by intracellular and extracellular enzymes (d), The temperature was 30°C, and the initial concentration of crude oil, NAP, PHE and PYR was 20 mg/L, shaking at 120 r/min, respectively

To disclose the degradation mechanism of PHs by strain RFC‐1, intra‐ and extracellular enzymes were extracted and used for treatment of crude oil, NAP, PHE, and PYR. Degradation efficiency of crude oil, NAP, PHE, and PYR by intra‐ and extracellular enzyme solutions followed the order of NAP > crude oil > PRY > PHE and increased over time in varying degrees. The diverse parts of the enzyme performed differently for the different substrates. From days 1 to 7, degradation efficiencies of crude oil, NAP, PHE, and PYR by the extracellular enzymes were slightly higher than those by intracellular enzymes (Figure 7a–d). The enzymes play an important role in removing PHs by fungi with stronger hydrocarbon‐degrading capacities than bacteria. Zhang et al. (2016) found that the extracellular enzymes of six *Aspergillus* strains isolated from oil‐contaminated soil are highly efficient in degrading crude oil, and showed strong abilities to degrade crude oil n‐alkanes into lighter fractions under oxygen‐deprived conditions. The extracellular enzyme of *Aspergillus fumigatus* also effectively metabolizes anthracene (Ye et al., 2011). Lipolytic enzyme of *Aspergillus niger* isolated from oil polluted soil was able to degrade PAHs in petroleum contaminated soil (Mauti, Onguso, Kowanga, & Mauti, 2016). Sandhu, Shakya, Deshmukh, Aharwal, and Kumar (2016) reported that the high degradation ability of *Aspergillus* sp. to the kerosene due to its outstanding mycelial growth and production of extracellular enzymes. Kadri et al. (2017) reported the enzymatic systems produced by fungi are keys to converting crude oil to carbon dioxide or into secondary metabolites. The fungal hydrocarbon‐degrading enzyme system is extracellular and unusually nonspecific (Zhang et al., 2016). However, Deive et al. (2009) found that the extracellular lipolytic activity is less than the intracellular enzyme activity. In the present study, degradation efficiency by extracellular enzymes of *Aspergillus* sp. RFC‐1 was higher than that by intracellular enzymes. Extracellular and intracellular enzymes may be contributed to PHs biodegradation.

## 4 CONCLUSION

*Aspergillus* sp. RFC‐1 can adsorb, absorb, and degrade crude oil and the three types of PAHs. Surface adsorption and cell absorption of crude oil, NAP, PHE, and PYR reached 43.9% and 35.7%, 50.5% and 67.7%, 59.0% and 83.9%, and 67.2% and 88.9%, respectively, and followed the order crude oil < NAP < PHE ≤ PYR. Adsorption content by heat‐killed mycelial pellets was constant after 40 min for crude oil and 10 min for PAHs; constant adsorption percentages of crude oil, NAP, PHE, and PYR by heat‐killed mycelial pellets amounted to 18.3%, 21.1%, 31.2%, and 38.3%, respectively. Biodegradation efficiencies of crude oil, NAP, PHE, and PYR totaled 51.8%, 84.6%, 51.3%, and 55.1%, respectively, within 7 days of incubation. Rapid accumulation (adsorption and absorption) and degradation of PHs by the strain RFC‐1 indicated its potential application in bioremediation of contaminated environments, including oil‐contaminated conditions. The intra‐ and extracellular enzymes of the strain RFC‐1 can effectively metabolize PHs, whereas extracellular enzymes play a more important role. This study can provide theoretical support for bioremediation of PHs pollutants.

## CONFLICT OF INTEREST

The authors declare that they have no conflict of interest.
