# Supplementary material for: Removal and biodegradation of different petroleum hydrocarbons using the filamentous fungus Aspergillus sp. RFC‐1
Source: Microbiologyopen. 2018 Mar 25;8(1):e00619. doi: 10.1002/mbo3.619 (PMC6341139; doi:10.1002/mbo3.619)
Supplement: Supplementary file 1 [file MBO3-8-e00619-s001.docx]

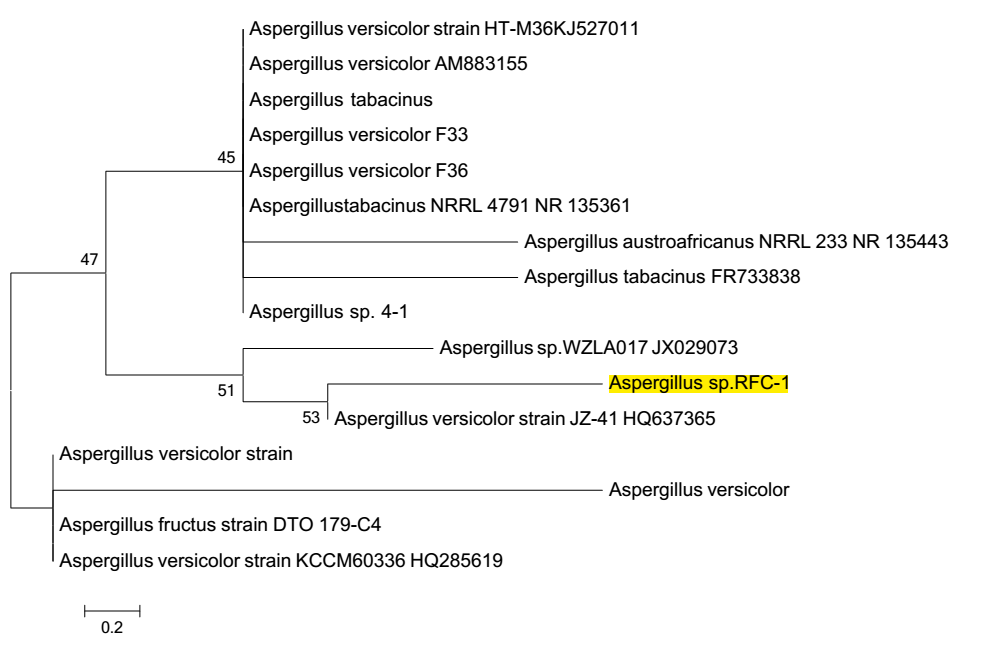


FGURE S1 Phylogenetic tree of *Aspergillus* sp. RC-1.

**TABLE S1**

Detection of biosurfactant production for the RFC-1 in MSM by oil spreading test

| Carbon source | Oil spreading (cm) |
| --- | --- |
| Crude oil | 4.7± 0.9 |
| NAP | 3.3± 0.7 |
| PEH | 3.8± 0.5 |
| PYR | 4.4± 0.4 |

Mean ± SD from triplicate determinations.
